# Modelling malaria pathogenesis

**DOI:** 10.1111/j.1462-5822.2008.01208.x

**Published:** 2008-10

**Authors:** Nicole Mideo, Troy Day, Andrew F Read

**Affiliations:** 1 Department of Biology, Queen's UniversityKingston, ON, Canada K7L 3N6; 2 Department of Mathematics and Statistics, Jeffery Hall, Queen's UniversityKingston, ON, Canada K7L 3N6; 3 Center for Infectious Disease Dynamics, Departments of Biology and Entomology, Pennsylvania State UniversityUniversity Park, PA 16802, USA

## Abstract

Almost 20 years after the development of models of malaria pathogenesis began, we are beyond the ‘proof-of-concept’ phase and these models are no longer abstract mathematical exercises. They have refined our knowledge of within-host processes, and have brought insights that could not easily have been obtained from experimentation alone. There is much potential that remains to be realized, however, both in terms of informing the design of interventions and health policy, and in terms of addressing lingering questions about the basic biology of malaria. Recent research has begun to iterate theory and data in a much more comprehensive way, and the use of statistical techniques for model fitting and comparison offers a promising approach for providing a quantitative understanding of the pathogenesis of such a complex disease.

## Introduction

Identifying factors that are involved in pathogenesis is important, but it is really only the first step towards a complete understanding of infectious disease. In the words of Ronald Ross, ‘To say that a disease depends upon certain factors is not to say much, until we can also form an estimate as to how largely each factor influences the whole result’ (cited by McKenzie, 2000). Ross was driven by this philosophy to find accurate mathematical models of malaria transmission and his efforts led to new insights into the biology of the disease and strategies for control. In particular, his quantitative analysis demonstrated that mosquito populations need not be eradicated, but rather need be driven only below a particular threshold in order to eradicate malaria. Subsequent implementation of malaria control measures validated these predictions (McKenzie, 2000) and, since then, numerous epidemiological models have been proposed and directed towards understanding various processes in malaria transmission.

**Fig. 1:**
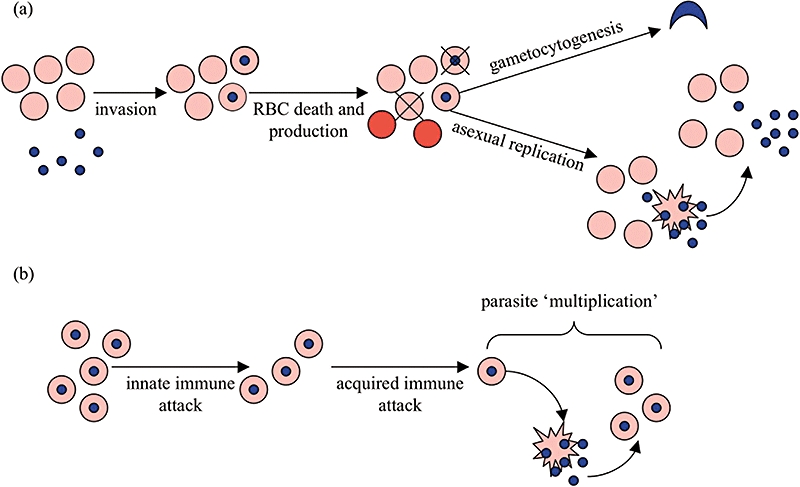
Schematic of two recent models of malaria pathogenesis. A. Modified from Mideo *et al*. (2008), this model tracks the densities of red blood cells (RBCs), merozoites and gametocytes. The main regulatory mechanism here is resource (i.e. RBC) abundance. B. The model of Dietz *et al*. (2006) focuses on the effects of innate and acquired immune responses and tracks the density of infected RBCs only. The abundance and action of different immune effectors is translated into probabilities of infected RBCs surviving their attack.

The interesting and important questions for the mathematical study of malaria are not, however, exclusively epidemiological, but span several levels of biological organization. Despite a large body of research on malaria pathogenesis (defined to be the within-host mechanisms through which the *Plasmodium* parasite causes disease), the relative significance of different factors in the development of disease is still debated (Miller *et al*., 2002). Research has focused mainly on two broad categories of factors: those that are resource-mediated [e.g. availability of red blood cells (RBCs), in which malaria parasites undergo asexual replication; Fig. 1A], and those that are immune-mediated (Fig. 1B). That we can categorize factors like this, however, in no way indicates that they are understood. Indeed, our resolution of the relative importance of these factors and of any interactions between them remains quite inadequate. For example, it is known that several immune components, like macrophages and natural killer cells, are likely involved in the innate immune response to malaria; but the interactions of these factors with acquired responses remain speculative, and conflicting experimental data leaves the role of many components in question (Stevenson and Riley, 2004). At best, we know that some factors are necessary for a particular outcome (e.g. interferon-γ and natural killer cells limit peak parasitemia, Stevenson and Riley, 2004), but the relative contribution of each factor to this outcome is still unclear.

In this article we follow Ross's philosophy, and suggest that, only when we can quantitatively predict the pattern of pathogenesis as a function of the underlying within-host regulatory factors can we legitimately claim to understand the processes at work. Although some might argue that the complexity of malaria biology puts this criterion beyond reach, we contend that such an objection is simply an acknowledgement of how little is known. As such, the development and testing of mathematical models of within-host processes is necessarily a vital component of research on malaria pathogenesis.

The value of having a sound, predictive, mathematical model of malaria pathogenesis cannot be overstated. Such models would allow *in silico* experiments of drug treatments and other interventions, thereby focusing efforts on targets that are likely to have the greatest impact. They would also allow for the evaluation of the too-often overlooked evolutionary consequences of treatments before they are made into policy. ‘No one dies of theoretical infections’ (McKenzie, 2000), which are also cheaper and quicker than animal experiments. Equally important, however, is the value of the whole process of developing such mathematical models. By developing models of pathogenesis, we are forced to confess our ignorance of many of the biological details of infection, and this alone can be useful for highlighting important areas for future empirical research. Mathematical models also remove all ambiguity in potential explanations for patterns of pathogenesis (there is no where to hide ignorance), and they clearly delineate the logical conclusions that stem from various hypotheses. If, for example, experimental inhibition of erythropoeisis suppresses parasite recrudescence during an infection, one might be led to suggest that RBC availability determines such recrudescence. But unless we can accurately predict such experimental changes in pathogenesis using a mechanistic model, our understanding of the processes involved is still incomplete. A mutual feedback between model development/testing and empirical (ideally experimental) research is necessary to develop this level of understanding.

The same mathematical tools that have become (almost) ubiquitous in studies of malaria epidemiology have the capacity to be equally informative when applied to questions of malaria pathogenesis. Yet modelling the within-host dynamics of malaria is a comparatively new practice, beginning just 20 years ago (e.g. Anderson *et al*., 1989). But unlike population dynamical models, which can rarely be fitted to truly replicated populations, within-host models can be very stringently challenged with data from numerous hosts. For the brave, this should make for much more rapid progress than is possible in epidemiology alone. Indeed, it may be no accident that the advent and expansion of in-host malaria models coincided with the explosive interest in mathematical modelling of within-host dynamics of HIV, where interactions between theorists and empiricist yielded considerable insights (see examples in Perelson and Nelson, 1999).

## Modelling approaches to malaria pathogenesis

The types of mathematical models of malaria pathogenesis we discuss in this review are based on a mechanistic description of the underlying biology of the system. This contrasts with purely statistical (i.e. curve-fitting models), although our exclusion of this class of models is not a value judgement. Indeed, such statistical models have also provided important insights (see Paul *et al*., 2007 for a recent example). Also, note that we do not restrict our discussion to models of human malaria. Much of the current theory is aimed at explaining experimental malaria infections in model organisms and, in particular, mice from which there is a wealth of data, and therefore we include these models as well.

A basic model of the dynamics of malaria infection would track the changes in density (e.g. number per microlitre) of the kinds of cells and/or molecules thought to be important in pathogenesis. For example, it might track the density of asexual and sexual parasite forms, red blood cells, and various types of immune effectors. As factors are identified as being potentially important in regulating these variables, they are translated into a mathematical formulation that can be incorporated into the basic model structure.

The ultimate aim when building models of malaria pathogenesis is to simplify the highly complex biological processes occurring during an infection into a comprehensible mathematical system from which inferences and predictions can be drawn. Critical in this process is the recognition that not all details of the biological system are relevant for understanding and predicting pathogenesis. Models need not (in fact should not) incorporate everything that we know about the biological system: we seek to understand the important components of reality, not to replicate the reality we do not understand. Indeed, the true power of a good model lies in its ability to expose the central agents responsible for the biological patterns under investigation by dispensing with the irrelevant details. Models help us to determine what is irrelevant.

To illustrate what such models typically look like and how, through the process of model development, the irrelevant biological details are uncovered, we present a simplified generic example based on Mideo *et al*. (2008). The model is in discrete time to account for the distinctly discrete life cycle of malaria parasites, and the densities are evaluated every day, corresponding to the 24 h cycle of the rodent malaria system on which this model was based. The model predicts how the density of three quantities, merozoites (*M*), gametocytes (*G*), and red blood cells (*R*), change from one day to the next;

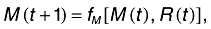


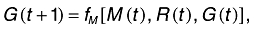


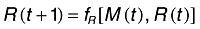

The above equations capture the idea that the density of each quantity in the next day (time *t* + 1) is some function of their densities on the present day (time *t*). Notice that two of these functions do not depend on the gametocyte density, *G*(*t*), reflecting an assumption that gametocytes play no role in determining the merozoite or RBC counts on the next day. Other assumptions about how various biological processes work (e.g. erythropoeisis, RBC infection, gametocytogenesis, etc.) are captured by the specific forms of the functions *f*_*M*_[*M*,*R*], *f*_*M*_[*M*,*R*,*G*] and *f*_*R*_[*M*,*R*] (Table 1).

**Table 1:** Example mathematical descriptions of different underlying assumptions.

Equations/variations	Assumptions
Merozoite density	
*M*(*t* + 1) = *ba*[*M*(*t*)]*R*(*t*)(1 − *g*)	• A proportion of susceptible RBCs becomes infected. This proportion is described by a function, *a*[*M*(*t*)], which depends on the density of merozoites. Infected RBCs produces *b* daughter merozoites. Asexual replication occurs in a proportion, 1 − *g*, of all infected RBCs.
*M*(*t* + 1) = *ba*[*M*(*t*)]*R*(*t*)(1 − *g*)*e*^*−cT*(*t*)^	• As above but with an immune response as well. The probability of an infected RBC surviving immune attack is given by *e*^*−cT*(*t*)^, and decreases with increasing immune cell density. The parameter, *c*, describes the susceptibility of infected RBCs to immune attack.
Red blood cell density	
*R*(*t* + 1) = *θ* + *R*(*t*) − *a*[*M*(*t*)]*R*(*t*)	• A constant number, *θ*, of RBCs (per microlitre) are produced daily. There is no natural death of RBCs; only loss is through infection.
*R*(*t* + 1) = *s*[*R*(*t*)] + *R*(*t*) − *a*[*M*(*t*)]*R*(*t*)	• A more general model in which daily RBC production, *s*, is an arbitrary function of current RBC density.
*R*(*t* + 1) = *s*[*R*(*t* −*τ*)] + *R*(*t*) − *a*[*M*(*t*)]*R*(*t*)	• As above, but now daily RBC production is time-lagged to account for the maturation time of RBC precursors (production is a function of RBC density *τ* days earlier).
Gametocyte density	
*G*(*t* + 1) = *G*(*t*) + *ga*[*M*(*t*)]*R*(*t*)	• A proportion, *g*, of all infected RBCs produce gametocytes.
*G*(*t* + 1) = *G*(*t*) + *ga*[*M*(*t*)]*R*(*t*) − *dG*(*t*)	• As above but a proportion, *d*, of gametocytes decay each day as well.
*G*(*t* + 1) = *G*(*t*) + *ga*[*M*(*t* − *τ*)]*R*(*t* − *τ*)	• As in the first model, but gametocytes are sequestered for *τ* days before maturing and being released into the bloodstream.
Immune cell density	
*T*(*t* + 1) = *T*(*t*) + *hT*(*t*)	• Immune cell densities increase exponentially. Each immune cell activates *h* others. Immune cell activation does not require contact with infected cells or merozoites.
*T*(*t* + 1) = *T*(*t*) + *ja*[*M*(*t*)]*R*(*t*)	• Production of immune cells is proportional to infected RBC density.

For each of merozoite density (*M*), RBC density (*R*), gametocyte density (*G*) and immune cell density (*I*), two or three different hypotheses of increasing complexity are presented.

Models like that above can also be further refined as necessary, by including things such as RBC age structure and time-lags in erythropoeisis. They can also be extended to include other regulatory factors related to innate and adaptive immune responses. For example, one might introduce other variables that represent the densities of different immune effectors molecules and cells. If we use *T*(*t*) to denote the density of specific T cells on day *t* then the model might be extended as

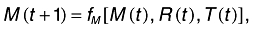


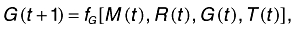


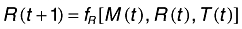


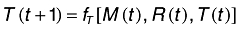

where the functions *f*_*M*_[*M*,*R*,*T*], *f*_*G*_[*M*,*R*,*G*,*T*], *f*_*R*_[*M*,*R*,*T*] and *f*_*T*_[*M*,*R*,*T*] are specified to account for the relevant assumptions about how these processes work (Table 1). The predictions of each model obtained by employing a different set of assumptions can then be tested against data to determine its ability to explain known patterns of pathogenesis (Mideo *et al*., 2008).

Each model, with its associated assumptions about how the important regulatory factors come into play, represents a different biological hypothesis about what determines malaria pathogenesis (Johnson and Omland, 2004). It might well turn out that a given model does not accurately reflect the biology of the system, but if so, something new about the biology and the importance of various regulatory factors would thereby have been learned. The clarity that comes from having made such unambiguous assumptions often will also point towards new empirical questions that need to be answered.

Consider again, the recrudescences in parasite density that occur during some malaria infections. One explanation for these peaks is that they are the result of antigen switching and immune escape by the parasite (Brown and Brown, 1965; Phillips *et al*., 1997). That secondary peaks are antigenically distinct does not, however, mean that the peaks exist *because* they are antigenically distinct, and an alternative explanation is that the peaks simply track resource availability – parasite densities increase as red blood cell densities rebound from the destruction wrought by the initial wave of parasites. An experimental approach to testing these hypotheses might alter an immune component of a model organism, generate malaria infections, and then see if these peaks still occur. Deciding what immune component to alter, when to alter it, and by how much, however, makes this line of investigation non-trivial.

A modelling approach to this question can provide two valuable kinds of information. First, as is often done in physics, mathematical simulations of experimental procedures can help to inform researchers which manipulations, of the vast array of possibilities, are likely to be the most informative. As with the case of RBC availability mentioned earlier, ultimately we cannot claim to understand the processes that are occurring unless we can accurately predict the outcome of such experimental manipulations.

Second, mathematical models can provide inferential power. By building models that include or exclude antigen switching mechanisms and immune responses, we can determine the conditions under which secondary parasite peaks are predicted to occur and if these regulatory factors are relevant for this particular pattern. In fact, models have revealed that both resource availability (e.g. Hetzel and Anderson, 1996; Mideo *et al*., 2008) and certain forms of antigen switching (e.g. Paget-McNicol *et al*., 2002) are plausible explanations for parasite recrudescences. A more thorough modelling approach that incorporates each of these processes, in isolation and in combination, will then help to resolve this issue, and inform future experiments. Below, we discuss how statistical model selection techniques are increasingly being used to compete multiple models and test alternative hypotheses.

## Insights

Mathematical models of malaria pathogenesis have helped develop our knowledge in many ways. Due to an increasing interest in this approach, we cannot cite all important findings, and instead we therefore focus on a few developments that reflect the range of applications of these models.

### Effective immune targets

One area that has received a lot of attention is host immune responses to malaria. Despite our incomplete knowledge of these processes (as described above), models have helped elucidate some of their general characteristics. Models of malaria have repeatedly demonstrated that immune responses are more effective if directed towards infected RBCs rather than free-living merozoites (Anderson *et al*., 1989; Hetzel and Anderson, 1996; Haydon *et al*., 2003). This is perhaps not surprising given the short lifespan of merozoites in the bloodstream (on the order of minutes), but tackling this question theoretically allows for the quantification of this difference in efficacy (Haydon *et al*., 2003). These results have obvious implications for vaccine design.

### Protective immune control

Also, as we would expect, innate immune responses are predicted to be most important during initial parasite peaks and progressively less important throughout the course of infection (Molineaux *et al*., 2001). Here, Molineaux and colleagues have actually quantified the *relative* importance of three types of immune responses (innate, acquired variant-specific and acquired non-variant-specific) over successive parasite peaks, finding, for example, that innate immune responses are almost entirely responsible for controlling the primary peak but are completely absent after the sixth peak.

The approach of Molineaux and colleagues is also unique in that they estimate model parameters for several individual host data sets, allowing variation in parasite traits between variants and between hosts. This results in some novel, testable, predictions. In particular, to recover differences in patterns of infection among hosts, the rank order of the parasite variants' baseline multiplication factors differed between hosts (i.e. the fastest growing variant in one host was not the fastest growing in all hosts, Molineaux *et al*., 2001). While the authors suggest this might not be a biologically justified conclusion, such a variant by host interactive effect on parasite growth rate could be tested experimentally with a model system.

### Clonal interactions

Immune responses will also have effects on the competition between parasite clones within a host. Hellriegel (1992) showed that competitive suppression of a superior clone, via a common immune response, was possible if the inferior clone arrived first (i.e. several days earlier). This prediction has since been tested and validated with experimental infections in mice (de Roode *et al*., 2005). The paradoxically low numbers of transmissible parasite forms in malaria infections (Taylor and Read, 1997) can also be explained by competition mediated by a shared immune response (McKenzie and Bossert, 1998). Instead of producing multiple daughter merozoites (each with the potential to infect another RBC), a small fraction of infected RBCs produce transmissible gametocytes. A common immune response that both was elicited by, and that targeted merozoites, would favour parasite clones that could quickly build up a ‘stock’ of merozoites during competition, i.e. those with low levels of conversion to gametocytes (McKenzie and Bossert, 1998). Competition for access to red blood cells may also be sufficient for generating selection for low levels of conversion to gametocytes (Mideo and Day, 2008). In the time frame of a single infection, there is experimental evidence that parasites alter gametocyte sex ratios in response to co-infection (Reece *et al*., 2008) and alter rates of conversion to gametocytes in response to drug treatment (Buckling *et al*., 1999), however, adjustment of conversion rates in response to co-infection has not yet been demonstrated (Wargo *et al*., 2007). Further investigation of a potentially plastic response is warranted, as is a comparison of levels of conversion in *Plasmodium* species that have been exposed to different amounts of co-infection over an evolutionary time frame (Mideo and Day, 2008).

### Gametocytogenesis

Models of human malaria, fitted to data from malariatherapy patients, have shown that the level of conversion to gametocytes changes significantly during the course of infection (Diebner *et al*., 2000; Eichner *et al*., 2001). However, the pattern of the shift in conversion rates remains unexplained. Several factors have been identified as likely influencing gametocyte production (Dyer and Day, 2000; Talman *et al*., 2004), and incorporating these into models could help in understanding the dynamics of gametocytogenesis.

### Consequences of RBC preference

In humans, parasite preference for certain ages of RBCs seems to relate to disease severity. The most severe disease is caused by *P. falciparum*, which infects RBCs of all ages. In contrast, less deadly species show preferences for infecting either younger (reticulocytes) or older RBCs (Paul *et al*., 2003). These cell preferences have been incorporated into models of human malaria, where they have been shown to have a significant effect on infection dynamics, and also help to explain the differences in clinical observations between species (McQueen and McKenzie, 2004). Recent models have explored whether this sort of cellular tropism is an important determinant of the dynamics of different rodent malarias. For example, a strong preference of *P. berghei* for reticulocytes can explain prolonged low levels of circulating reticulocytes that tend to be thought of as the result of suppressed RBC production (Cromer *et al*., 2006).

In contrast, *P. chabaudi* is generally thought to indiscriminately infect RBCs of all ages, but parameter estimates from a recent model show that parasites invade older RBCs at a rate that is an order of magnitude higher than for reticulocytes. Despite this, parasites did better in reticulocytes, producing more daughter merozoites per infected cell (Mideo *et al*., 2008). This effect, however, appears to be clone-specific which suggests it might be a mechanism that explains different levels of virulence between clones. Another model allows for *P. chabaudi* parasites to further discriminate between ages of RBCs and predicts that the size of the age range a particular clone can infect is correlated with its virulence (Antia *et al*., 2008). Each of these predictions can (and should) be tested empirically.

### Manipulation of erythropoeisis

The idea that erythropoeisis is suppressed during malaria infection has been refuted by models of both human (Jakeman *et al*., 1999) and rodent (Mideo *et al*., 2008) malaria. In fact, during periods of disease-induced anaemia, these models predict that hosts increase RBC production well above baseline rates. Intriguingly, models fitted to data from experimental *P. chabaudi* infections suggest that host response to anaemia varies depending on the genotype of the infecting parasites (Haydon *et al*., 2003; Mideo *et al*., 2008). Whether this effect can also help explain different levels of virulence between parasites clones remains unclear, as in one case the more virulent clone induced faster rates of RBC production (Haydon *et al*., 2003) and in the other, the opposite was true (Mideo *et al*., 2008). Unlike in Haydon *et al*. (2003), the data in Mideo *et al*. (2008) came from CD4^+^ T cell-depleted mice, indicating a possible interaction between parasite genotype and host immune status. Regardless of the relationship between virulence and erythropoeisis, the prediction of a clone-specific effect on this host trait could not likely be made from looking at data alone.

## Theoretical developments

Below we discuss criticisms of previous models of malaria pathogenesis and the improvements both realized in recent work and hoped for in the future. It should be noted that the issues we highlight are not malaria-specific; rather they really represent challenges to all modellers of disease dynamics.

### Biological realism

The best level of mathematical abstraction for any biological process should be determined by the nature of the question being addressed. For example, if one wants to identify mechanisms that can plausibly explain dynamics, schematic models are often sufficient. Many of the insights discussed above come from model approaches of this type. However, many of these same models have been criticized for their lack of realism (Molineaux and Dietz, 1999) and if one wants to use a model to make quantitative predictions about the dynamics of malaria pathogenesis, then a key goal is realistically capturing as much of the biology of the system as is necessary to explain data. In particular, early models of malaria pathogenesis allowed for no individual variation between hosts or parasites and failed to account for the distinctly discrete life cycle of malaria parasites (Molineaux and Dietz, 1999). The field is maturing and recent work has addressed these concerns (e.g. Molineaux *et al*., 2001; Dietz *et al*., 2006; Mideo *et al*., 2008), hopefully broadening the appeal (and thus the impact) of theoretical approaches for studying malaria pathogenesis.

Clearly, there is still some way to go regarding the mathematical capture of immunological processes. A major future challenge is to move beyond the mathematical caricatures of immune control (e.g. arbitrary functions, the grouping of distinct populations of immune effectors into ‘immune cells’) towards mathematical descriptions that more closely accord with the qualitative picture emerging from experimental immunology (e.g. Stevenson and Riley, 2004). Indeed, we expect mathematical models to play a critical role in identifying the key processes involved, and determining their relative importance.

### Tying theory to data

**Fig. 2:**
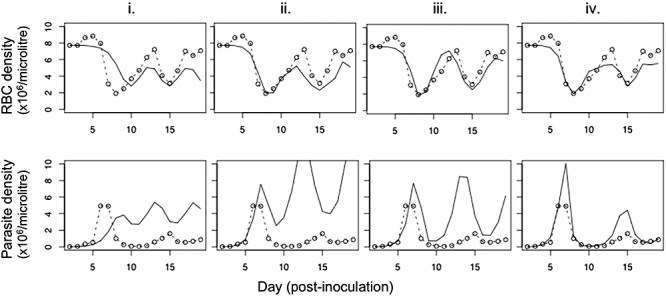
Model selection and validation. Data from a single CD4^+^ T cell-depleted mouse (dashed lines and dots) and predictions from four models (solid lines) Top panels, RBC densities; bottom panels, parasite densities. Model predictions are from four models representing different hypothesis about what regulates the dynamics of pathogenesis: i. no RBC age structure or parasite cell age preference and constant erythropoetic response; ii. no RBC age structure or parasite cell age preference and variable erythropoetic response; iii. RBC age structure, possible parasite cell age preference and constant erythropoetic response; iv. RBC age structure, possible parasite cell age preference and variable erythropoetic response. Models iii and iv provide statistically significantly better fits to the RBC data than models i and ii. As the models were fit only to the RBC data, the parasite data provide a means of model validation. It is clear that model iv is better than iii at qualitatively capturing the parasite dynamics. Model iv is selected as the ‘best’ model among those tested. See Mideo *et al.* (2008) for further details.

Another significant criticism of many of these models is that, while they often include some indication of the qualitative agreement between model predictions and experimental or clinical data, they tend to lack an investigation into whether alternative models could do as good a job, and whether the model predictions provide a statistically good fit for the data (Molineaux and Dietz, 1999). More rigorous approaches to model design, fitting and selection can help to resolve conflicts between current results. As a starting point, many theoretical studies do consider multiple biological hypotheses for what regulates malaria pathogenesis. Ideally, these should be translated into mathematical descriptions (i.e. models) of the hypotheses, and then the best model should be chosen from this pool of potential descriptions. For example, a resource-based model may capture the details schematized in Fig. 1A; a competing model, the immune regulation of Fig. 1B. Statistical procedures exist to compare the fit of each model's predictions to data using one of a number of different information-theoretic techniques (Hilborn and Mangel, 1997; Burnham and Anderson, 2002; Johnson and Omland, 2004; see Diebner *et al*., 2000; Mideo *et al*., 2008 for examples); we give an illustrative example in Fig. 2. Once a best model is chosen, determining whether it indeed does provide an acceptable *quantitative* description of observed dynamics requires further assessment. This kind of ‘goodness-of-fit’ analysis is still relatively uncommon in mathematical treatments of malaria, but recent research has begun to take this approach (Molineaux *et al*., 2001; Dietz *et al*., 2006; Mideo *et al*., 2008).

Finally, models ought to be validated by altering some model component, generating new predictions, and then empirically testing these with independent experiments. The experimental manipulations required to do this are only ethically feasible in model organisms. While there is still debate about the relevance of these experiments for human malaria (see Dietz *et al*., 2006), the fact that we can use them to test and refine theory highlights the utility of using model malaria systems. Further, if we cannot design theory that accurately captures the dynamics of malaria pathogenesis in a highly controlled setting (like clonal infections in inbred laboratory mice), there is no hope for a full understanding of more heterogeneous human malaria infections.

Model validation is an extremely important step in the theoretical process and even ‘bad’ validation can lead to new insights. For example, a recent model based on RBC limitation as an explanation for the dynamics of single-clone malaria infection in CD4^+^ T cell-depleted mice could not accurately capture all of the dynamics seen in experiments involving co-infection with two clones (Mideo *et al*., 2008). This suggests the existence of a hitherto unknown specific immune response that is independent of CD4^+^ T-cell control.

This example helps us illustrate two key points. First, collaboration between experimentalists and theorists is essential and can yield mutually beneficial insights. Second, particularly when data and theory are tied in this way, models that generate predictions that fail to accurately capture real observations can still be extremely informative.

## Future directions

An important goal of building mathematical models of malaria pathogenesis is to use them to evaluate interventions. By translating the mode of action of an intervention into changes in particular model parameters, the effects on disease progression within a host can be predicted. Recently, Dietz *et al*. (2006) took this approach to explore the effects of vaccination by altering several different model parameters corresponding to different (plausible) modes of vaccine action. They determined that the outcome of infection after vaccination is strongly host-dependent, and that certain types of vaccines are better at protecting against severe versus mild malaria. These results suggest that current methods of evaluating vaccines may be inappropriate (Dietz *et al*., 2006).

Changes to within-host parameters due to interventions will have influences on processes at higher levels of biological organization. Simply put, epidemiological processes like transmission are determined by parasite densities within hosts, which are regulated by all the factors we have discussed above and, likely, many more. Interventions like drug treatment and vaccination will alter within-host regulatory factors with effects that will scale up from the within- to the between-host level, influencing, for example, disease prevalence.

One way to account for these interactions is by using a theoretical framework that nests levels of biological organization. A few recent models have taken this approach and combined models of the within-host and transmission dynamics of malaria (McKenzie and Bossert, 2005; Mideo and Day, 2008). The combined model of McKenzie and Bossert (2005) leads to some novel predictions about how within-host details affect host population level processes. For example, they predict that increased antigenic diversity in a parasite population can lead to increased persistence of individual parasite genotypes. This is explained by the action of innate immune responses during superinfections, which allow recovery from secondary infection by a particular genotype before acquired responses (specific to that genotype) can build up, maintaining a pool of hosts susceptible to that genotype. Another finding of McKenzie and Bossert (2005) is that the number of hosts infected in their model is insensitive to changes in within-host gametocyte survivorship, as only below a certain threshold density does the actual number of gametocytes (versus presence) seem to have an effect on transmission success. Given these results, any intervention targeting this parasite stage ‘would need to be astonishingly effective’ (McKenzie and Bossert, 2005) to be of any consequence.

Although the aim of McKenzie and Bossert (2005) was not to evaluate interventions, nested models can be directed at this purpose as we know that changes to within-host parameters (as a result of, say, vaccine action) will have an effect on higher level processes. For example, nested models have demonstrated the potential for unintended, negative consequences in response to vaccination, including selection for increased virulence, depending on the specific vaccine target (e.g. Gandon *et al*., 2001; Ganusov and Antia, 2006) or vaccine coverage (e.g. André and Gandon, 2006). However, these are highly generalized models with non-disease-specific mathematical descriptions of within-host processes. As we have argued above, if our aim is to make quantitative predictions about outcomes for a specific disease, we ought to use strongly supported, biologically based models. Improving within-host models of malaria pathogenesis and combining these with epidemiological models will lead to better predictions about the effects of interventions.

Some of the potential evolutionary effects of interventions have been studied with model organisms and while their results are important they are not always well understood. Why, for example, does passaging malaria parasites through immunized mice result in selection for more virulent parasites (Mackinnon and Read, 2004)? In particular, what is the mechanism of this increased virulence, i.e. on what trait is selection acting? These empirical results may have serious implications for human health policy, yet these questions remain unanswered. With a good model of mouse malaria (one that has been derived from, calibrated and validated with data) we can replicate this experiment *in silico* with the aim of predicting what kinds of malaria parasites (e.g. those that undergo rapid antigen switching, replicate at higher rates or infect RBCs at faster rates) have an advantage in immunized hosts. Theory offers an easier and powerful approach for teasing apart mechanisms.

## Conclusions

The fundamental goal of any study of malaria pathogenesis is to bring new insights towards developing successful treatment and control measures for this disease. Given the lack of progress in the past, our best hope for tackling the problem of malaria is through a more comprehensive understanding of the mechanisms that determine its pathogenesis. Unless these processes can be translated into mathematical models that accurately capture the dynamics of pathogenesis, this understanding will remain out of reach. In the process of striving for a mathematical account of malaria pathogenesis, we will likely discover the existence of new regulatory factors and that some regulatory factors are largely unimportant. By determining the relative importance of what does matter, and how those factors interact, we will be able to predict the likely consequences of clinical interventions, such as vaccines and chemotherapeutic agents targeted at particular parasite stages, and novel interventions aimed at host factors which determine disease (immunopathology). Achieving this promise requires the careful interactions between experimental biologists who appreciate that useful models need not include every last detail of every pathway, and biomathematicians who are prepared to tackle the jargon, the huge experimental literature and the fuzzy uncertainties of real experimental data.
